# Imaging of conditional gene silencing *in vivo* using a bioluminescence-based method with thermo-inducible microRNAs

**DOI:** 10.1038/s41598-018-22932-3

**Published:** 2018-03-16

**Authors:** Karine Pinel, Coralie Genevois, Christelle Debeissat, Franck Couillaud

**Affiliations:** 1 Molecular Imaging and Innovative Therapies (IMOTION), EA7435, Bordeaux, 33000 France; 2 Present Address: Institute of Cardiovascular and Medical Sciences, BHF Glasgow Cardiovascular Research Centre, Glasgow, G12 8TA United Kingdom

## Abstract

RNA interference (RNAi)-based gene therapy has great potential in cancer and infectious disease treatment to correct abnormal up-regulation of gene expression. We show a new original method uses synthetic microRNAs combined with a thermo-inducible promoter to reduce specific gene expression. The targeted gene is the *luciferase firefly* reporter gene overexpressed in a subcutaneous tumor which allows the RNAi monitoring by bioluminescence imaging (BLI). The inducible inhibition was first demonstrated *in vitro* using genetically modified cells lines and then *in vivo* using the corresponding xenograft model in mice. Achieving spatio-temporal control, we demonstrate the feasibility to induce, *in vivo*, a specific gene inhibition on demand. Future applications of this RNAi-based gene therapy, which can be restricted to pathological tissue, would offer wide-ranging potential for disease treatment.

## Introduction

RNA interference (RNAi) is an evolutionarily-conserved mechanism which has become a revolutionary strategy to regulate gene expression. This biological process, mediated by small interfering RNAs (siRNAs), short hairpin RNAs (shRNAs) or microRNAs (miRNAs), has rapidly emerged as a key experimental tool for gene function analysis and target validation in mammalian systems, both *in vitro* and *in vivo*. As a consequence, RNAi appears to be an attractive technology for gene therapy to down-regulate specific genes^1^. However, RNAi induction mediated by siRNAs is limited by their pharmacokinetic properties, their poor cell penetration and their limited activity *in vivo*^2^. Similarly, although not a prerequisite, experimental studies often use polymerase III-dependent promoters for applications that require shRNA synthesis. The development of shRNA embedded in a miRNA scaffold driven by RNA polymerase II makes tissue-specific or inducible RNAi possible by using a wide variety of promoters. Furthermore, biosynthesis of optimized shRNAs based on miRNA cell processing machinery provide efficient and safe therapeutic routes for RNAi induction *in vitro* and *in vivo*^3–5^. MiRNAs are a class of non-coding RNA implicated in the regulation of around 30% of the cell transcriptome^6^. MiRNAs can naturally direct gene silencing at a post-transcriptional level. Most gene therapies require restriction of the treatment to the targeted tissues to minimize unwanted side effects. Thus, RNAi-based therapies require spatial control of the induction. Heat-sensitive transgene expression systems have been proposed for use in gene therapy to enable spatial control of gene activity, also offering temporal control. HSP (heat shock protein)-related promoters are inducible and external heat treatment enables their activation. *In vitro* feasibility of the transcription of an artificial miRNA by HSP-related promoters has been reported^7^ but only following transient cell transfection. However, the Hsp70B promoter has many advantages including its low basal activity and the heat-induced expression^8–11^ which could create new approaches for Hsp-controlled RNAi systems *in vivo*. Moreover, the efficient control of gene expression *in vivo* using a HSP-related promoter has been reported using encoding genes such as *HSV thymidine kinase*, *FAS ligand* and *cytokine* genes^9,12–14^ or reporter genes for *in vivo* monitoring^10,15,16^.

In this study, we propose a new *in vivo* method to combine the shRNA embedded in a miRNA scaffold inhibitory effect with its thermo-induced expression driven by the Hsp70B promoter. This article demonstrates the feasibility and efficiency of this strategy *in vitro* followed by its implementation *in vivo* using the reporter gene *firefly luciferase* (*LucF*) as the target gene.

## Material and Methods

### Animals

Animal manipulations were performed in agreement with both European and French directives. The present project was approved by the local ethical committee (CEEA 50) under agreement A50120194. Female immunodeficient NOG (NOD/SCID/IL-2Rγ^null^) mice (6- to 10-weeks-old) were purchased and reared at the University of Bordeaux animal facilities. Animals were maintained in standard conditions under a 12 hour light/dark cycle with water and food provided *ad libitum*. Animals were anesthetized with 2% isoflurane (Belamont, Nicholas Piramal Limited, London, GB) in air. The posterior region of the mice was shaved with clippers and a depilatory cream on the day before imaging.

### DNA constructs

Plasmids pcDNA_6.2_-GW EmGFP-miRLuc and pcDNA_6.2_-GW EmGFP-miRneg were purchased from Invitrogen (BLOCK-iT^TM^ system, Life Technologies^TM^, Carlsbad, CA, USA). These vectors allowed for co-cistronic expression of the EmGFP (Emerald Green Fluorescent Protein) reporter gene and synthetic miRNA under the transcriptional control of the constitutive CMV promoter. Correlation between EmGFP and miRNA expressions was previously demonstrated (Life Technologies^TM^). The pcDNA_6.2_-GW EmGFP-miRLuc encoded a mature synthetic miRLuc specific to the LucF mRNA. pcDNA_6.2_-GW EmGFP-miRneg encoded a mature miRNA called miRneg, unrelated to any known gene and used as a negative control. To increase efficiency of the inhibitory effect of miRNA, 3 additional copies of each pre-miRNA were cloned into the pcDNA_6.2_-GW EmGFP-miRLuc and pcDNA_6.2_-GW EmGFP-miRneg vectors to obtain the pcDNA_6.2_-GW EmGFP-4*miRLuc and pcDNA_6.2_-GW EmGFP-4*miRneg plasmids.

The Hsp70B promoter sequence was excised from pD3SX vector (Stressgen Biotechnologies Corp. Victoria, BC, Canada). *Renilla luciferase* (*LucR*) reporter gene was excised from pRL-CMV vector (Promega). By several cloning steps the CMV promoter from pcDNA_6.2_-GW EmGFP-4*miRLuc and pcDNA_6.2_-GW EmGFP-4*miRneg was replaced by a Hsp-LucR-Hsp cassette to obtain pcDNA_6.2_-Hsp-LucR-Hsp-EmGFP-4*miRLuc and pcDNA_6.2_-Hsp-LucR-Hsp-EmGFP-4*miRneg constructs.

### Cell culture and stable transfections

The different U87 cells lines were maintained in Dulbecco’s modified Eagle’s medium (DMEM; Life Technologies^TM^) supplemented with 10% fetal calf serum (Life Technologies^TM^), 1% antimycotic-antibiotic mix (PSA; Life Technologies^TM^) and 1% non-essential amino-acid (MEM NEAA; Life Technologies^TM^) at 37 °C and 5% CO_2_. Cells were subcultured 1:3 by trypsinization upon reaching 90% confluence. The stably transformed U87 CMV-LucF cell^17^ was further transformed by either pcDNA_6.2_-Hsp-LucR-Hsp-EmGFP-4*miRLuc or pcDNA_6.2_-Hsp-LucR-Hsp-EmGFP-4*miRneg using TransFast^TM^ transfection reagent (Promega, Madison, WI, USA) according to the manufacturer’s instructions. Stably transformed cells lines miRLuc and miRneg were selected with 2 µg/mL blasticidin (PAA, Piscataway, NJ, USA) and amplified for both *in vitro* experiments and tumor generation in NOG mice.

### *In vitro* luciferase assays

Firefly and *Renilla* luciferases enzymatic activities were measured on cells lysates (25,000 cells) using the Dual-Luciferase® Reporter Assay System (Promega) using a luminometer (LUMAT 9501; Berthold Technology, Bad Wildbad, DE). Light production was expressed in Relative Light Units (RLU).

### RNA isolation, reverse transcription and real-time quantitative polymerase chain reaction

#### RNA isolation

Total RNA and miRNA were extracted from tumors (40 mg) and cells with the miRNeasy Mini kit (Qiagen, Hilden, DE) according to the manufacturer’s instructions. During extraction, DNase digest is performed (RNase-Free DNase set; Qiagen). Total RNA was quantified using Quanti-iT™ RiboGreen RNA Kit (Invitrogen, Molecular probes) on a fluorimeter (Versafluor®; Bio-RAD).

#### Reporter gene qRT-PCR

Reverse transcription was performed on 1 µg total RNA using ImProm-II™ Reverse Transcription System (Promega, Madison, WI, USA) and random hexameric primers (Promega). Real time PCR reactions were performed with SYBR® Green I dye (Thermo Scientific, St. Leon-Rot, DE) on MyIQ Real Time thermocycler (Bio-RAD, Hercules, California, USA) according to manufacturer’s protocol and primers (Supplementary Table S1) (Eurogentec; Seraing, BE). Normalization was performed regarding to *36B4* gene level and the relative expression for a target gene was calculated using the comparative Ct method (2^−ΔΔCt^).

#### miRNA qRT-PCR

Stem-loop reverse transcription reaction is performed on total RNA (1 µg) using Multiscribe RT enzyme (TaqMan® MicroRNA Reverse Transcription kit; Life Technologies^TM^) according to manufacturer’s protocol and using the stem–loop RT primer (Eurogentec) (Supplementary Table S2). Real time PCR reactions were performed using SYBR®Geen I dye and specific primers (Supplementary Table S3). The expression level of miRNA was normalized to the small nucleolar RNA RNU44 and the relative expression was calculated using the comparative Ct method (2^−ΔΔCt^).

### Flow cytometry

Cells were trypsinized and washed in PBS. Each cell suspension in PBS was analyzed by flow cytometry to determine the level of EmGFP expression using a Guava easycyte^TM^ Flow Cytometer (Merck Millipore, Darmstadt, DE). For each analysis, 10,000 events were captured. Dual blue (488 nm excitation wavelength) excitation laser and green fluorescent channel (525/30 nm) were used to quantify the number of positives cells. The flow cytometry data was analyzed with the Incyte software.

### Subcutaneous tumor generation

U87 cell suspension (2 × 10^6^ cells; 100 µL PBS) was inoculated into the subcutaneous tissue in the mice using an U-100 insulin syringe (TERUMO®, Cottontail Lane Somerset, NJ, USA). MiRLuc and miRneg cell lines were injected into hind legs. For this, one tumor per animal was generated, using the left side for the miRLuc cell line and the right side for the miRneg cell line. Once the tumor became palpable, tumor width and length were measured using a digital caliper by the same researcher to prevent observation differences, during all the time-course. The tumor volume was then calculated using the Feldman *et al*. formula^18^: Volume = π/6 ∙ f ∙ (length ∙ width)^3/2^.

### Hyperthermia treatment

#### Cell hyperthermia

Cells were plated 48 hours before the experiment (15,000 cells per well into 4-well plates). Hyperthermia was induced using a water bath (45 °C; 20 min) and the media was renewed using pre-warmed media, then the cells were returned to the incubator at 37 °C and 5% CO_2_.

#### Tumor hyperthermia

When tumors reached a volume of 110 ± 80 mm^3^, anesthetized mice were placed on a polystyrene-isolated platform floating on the surface of a water-bath. Mice hind legs were dipped into hot water (45 °C +/− 0.1 °C; 8 min) by a hole in the platform, thus heating only the legs while the rest of the body was lying on the isolation material.

### *In vivo* bioluminescence imaging

BLI was performed at VivOptic (UMS 3767, Bordeaux University, FR) using a Lumina LT system (Perkin Elmer Inc., Boston, MA, USA) including a highly sensitive CCD camera. For LucF signal detection, mice received an intra-peritoneal injection of D-luciferin (Promega, Madison, WI, USA, 2.9 mg in 100 µL PBS) and were sedated 7 min later. For LucR signal detection, mice received an intra-peritoneal injection of ViviRen (Promega, 50.8 µg in 100 µL PBS-BSA 0.1%) and were sedated 17 min later. Bioluminescence images (1 min, 4 × 4 binning) and photographs (100 ms exposure) were taken at 10 min or 20 min after the substrate injection for LucF and LucR respectively. The bioluminescence signal was converted using a false color scale and images representing the spatial distribution of emitted photons were generated using Living Image software (Perkin Elmer Inc.) and superimposed on to the photograph. BLI analysis was performed semi-automatically by placing a small region of interest (ROI) on the leg. The mean light intensity (in photons.s^−1^.mm^−2^.sr^−1^) was measured within this ROI.

### Statistical analyses

All statistical analyses were performed using a two-tailed unpaired Student’s *t* test for comparison of two groups and a statistical difference was considered as *P < 0.05, **P < 0.01 and ***P < 0.001. In the qRT-PCR experiment, the statistical analyses were made on the dCt for more accuracy. All data were represented as mean ± s.e.m.

## Results and Discussion

### A thermo-inducible inhibition strategy through the Hsp70B promoter

**Figure 1:**
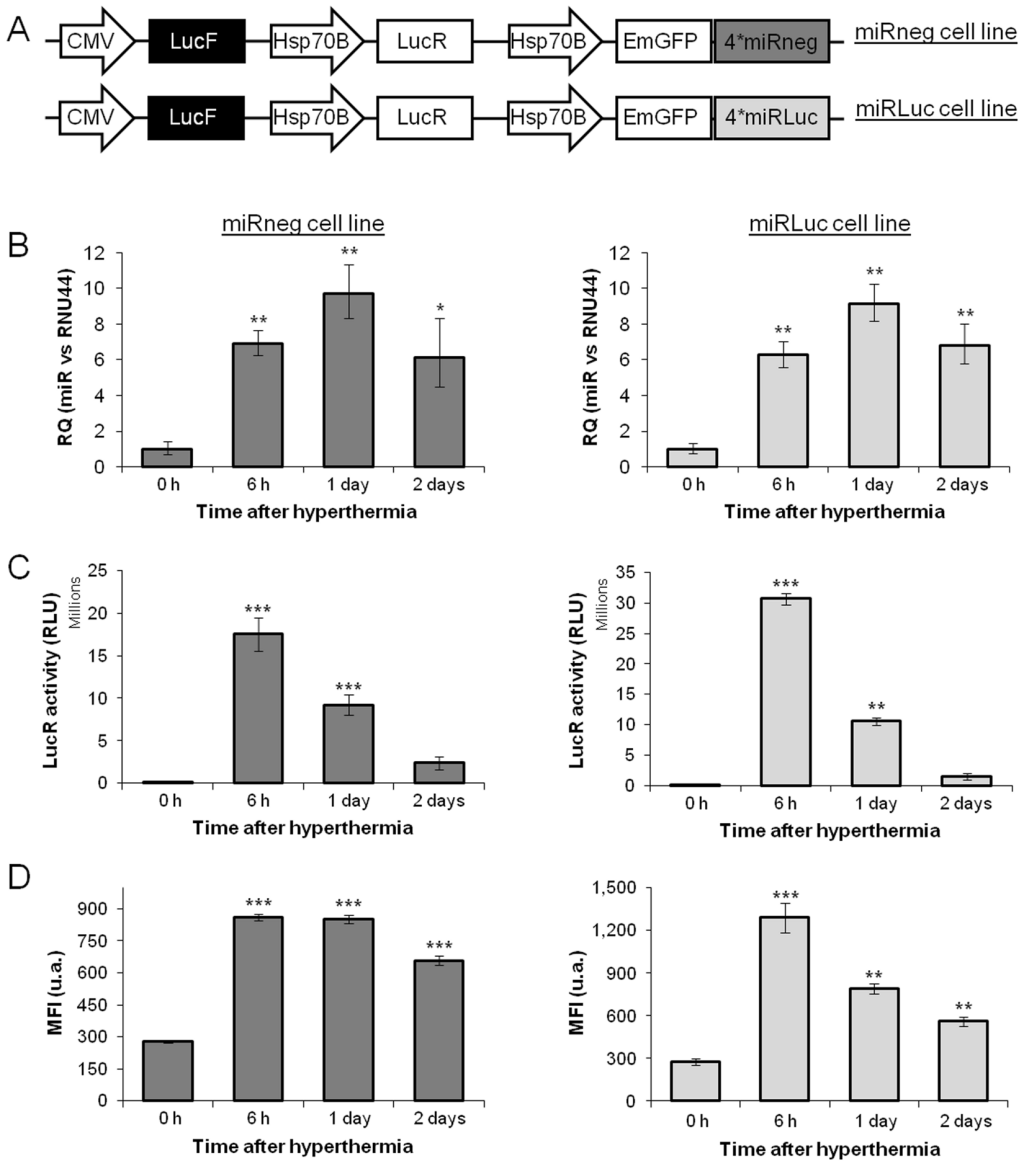
*In vitro* heat-shock efficiency. (**A**) Diagram of the constructs. (**B**) Relative quantification (RQ) of miRNAs in heat-shock induced (45 °C; 20 min) miRneg and miRluc cell lines respectively (n = 3) (Student’s t-test; *p < 0.05 and **p < 0.01: *vs*. 0 h). (**C**) Measurement of the LucR activity by *in vitro* enzymatic assay with a maximum at 6 h post-hyperthermia in both miRLuc and miRneg cell lines (n = 3) (Student’s t-test, **p < 0.01 and ***p < 0.001: *vs*. 0 h). (**D**) Mean fluorescence intensity (MFI) of EmGFP determined by flow cytometry with a maximum at 6 h post-hyperthermia in both miRLuc and miRneg cell lines (n = 3) (Student’s t-test, **p < 0.01 and ***p < 0.001: *vs*. 0 h).

To demonstrate the feasibility of our method, we generated cell lines which constitutively express the *LucF* reporter gene (CMV promoter) and contained inducible *Renilla luciferase* (*LucR*), *emerald GFP* (*EmGFP*) and 4 copies of synthetic miRNAs under the Hsp70B transcriptional control (Fig. 1A). This strategy aimed to control the heat-induction using LucR and EmGFP as reporters and monitoring the effect of the miRNA on the LucF expression *in vivo*. Furthermore, the separate monitoring of LucF and LucR expressions allowed the distinction between heat-induction and the effect of the synthetic miRNA as these luciferases have their own substrate and show distinct kinetics of light production *in vivo*. RNAi was assessed by specific synthetic miRNAs (miRLuc) targeting LucF mRNA (Supplementary Figure S4) and miRneg (unrelated to any known gene) was used as a negative control. After *in vitro* validation, we generated subcutaneous tumors in mice to follow heat-induced target gene inhibition by bioluminescence imaging (BLI), involving the non-invasive interrogation of living animals using light emitted from luciferase-expressing cells.

### Thermo-induced RNAi *in vitro*

**Figure 2:**
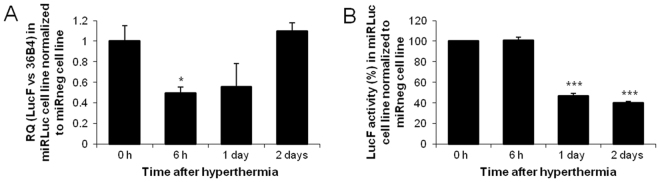
Thermo-inducible inhibition of LucF reporter gene *in vitro*. (**A**) Relative quantification (RQ) of LucF mRNA in miRLuc cell line normalized to miRneg cell line for each time point. The graph represents the RQ compared to 0 h time point (n = 3) (Student’s t-test; *p < 0.05: *vs*. 0 h). (**B**) LucF activity determined by *in vitro* enzymatic assay at different time points after hyperthermia. Values were normalized to miRneg cell line for each time point. The graph represents the percentage of LucF activity compared to 0 h time point (n = 3) (Student’s t-test; ***p < 0.001: *vs*. 0 h).

*In vitro*, after a heat shock of the miRLuc and miRneg cell lines, the synthetic miRNAs expressions showed an increase of miRLuc and miRneg miRNAs levels 6 hours after treatment (Fig. 1B). To control the Hsp70B promoter activation after a short and transient heat shock of miRLuc and miRneg cell lines, LucR and EmGFP expression were followed using enzymatic assays and flow cytometry respectively (Fig. 1C and D respectively). Our data shows low basal activity for LucR and EmGFP. A maximal level of activation was reached 6 hours after hyperthermia for the two reporter proteins in both cells lines, thus suggesting an early activation of Hsp70B promoters in accordance with the literature. Interestingly Fig. 1D showed a difference in EmGFP kinetics between cell lines which prompted us to not use this reporter as an internal control for the subsequent experiments. Additionally, we measured the endogenous Hsp70 pathway which showed a transient induction of Hsp70 mRNAs expression (transcripts resulting from the transcription driven by all the Hsp70 promoters) (Supplementary Figure S5). Although Hsp70B promoter was described to exhibit a relatively low basal activity, as shown in Fig. 1, our data demonstrated the efficiency of the Hsp70B-induced promoter activation by hyperthermia *in vitro*. This allowed us to pursue further investigations on miRLuc efficiency. We investigated whether miRLuc impacted on LucF mRNA content and LucF activity in the miRLuc cell line (Fig. 2A and B respectively, both normalized to miRneg cell line). Our results showed a 50% decrease in LucF mRNA content expression 6 hours following hyperthermic miRLuc expression induction by hyperthermia which was sustained for 24 hours before returning to normal levels two days post-treatment (Fig. 2A). This result showed the efficiency of the synthetic miRNA on the degradation of its mRNA target. Furthermore, outcomes of this repression were delayed in time for LucF activity. LucF activity showed a minimum 50% decrease one to two days following miRLuc induction by heat treatment (Fig. 2B). Overall, *in vitro* experiments validated the thermo-induced RNAi strategy.

### *In vivo* gene inhibition *via* Hsp70B-induced miRNA expression

**Figure 3:**
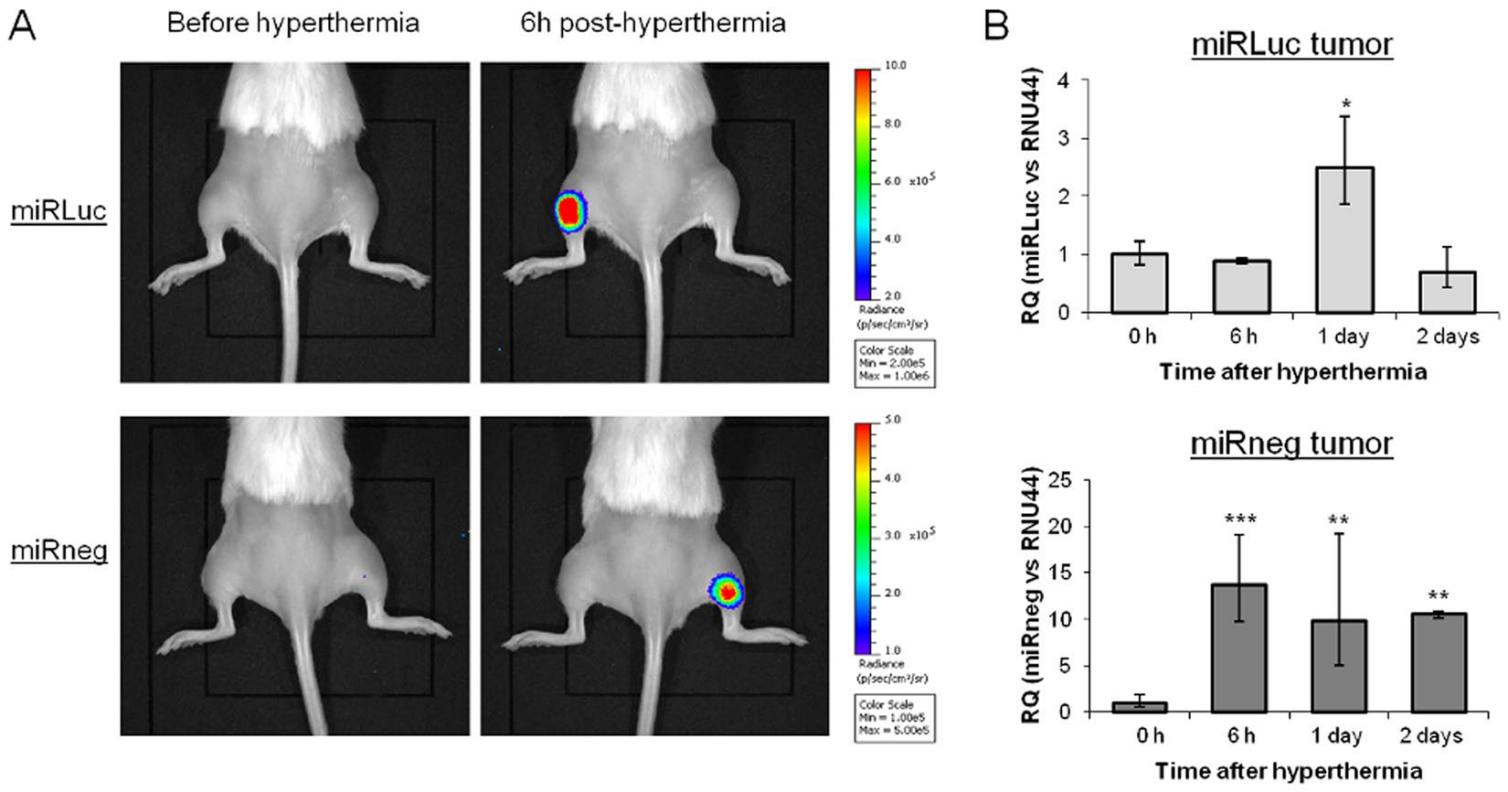
*In vivo* heat-shock efficiency. (**A**) Subcutaneous xenografts were generated by the injection of miRLuc or miRneg cell lines in mice hind legs. Representative image of LucR signal detection for miRLuc and miRneg tumor following intra-peritoneal injection of ViviRen. (**B**) Relative quantification (RQ) of miRLuc and miRneg from miLuc and miRneg tumors respectively determined by qRT-PCR at different time after hyperthermia (n = 3) (Student’s t-test, *p < 0.05, **p < 0.01 and ***p < 0.001: *vs*. 0 h).

**Figure 4:**
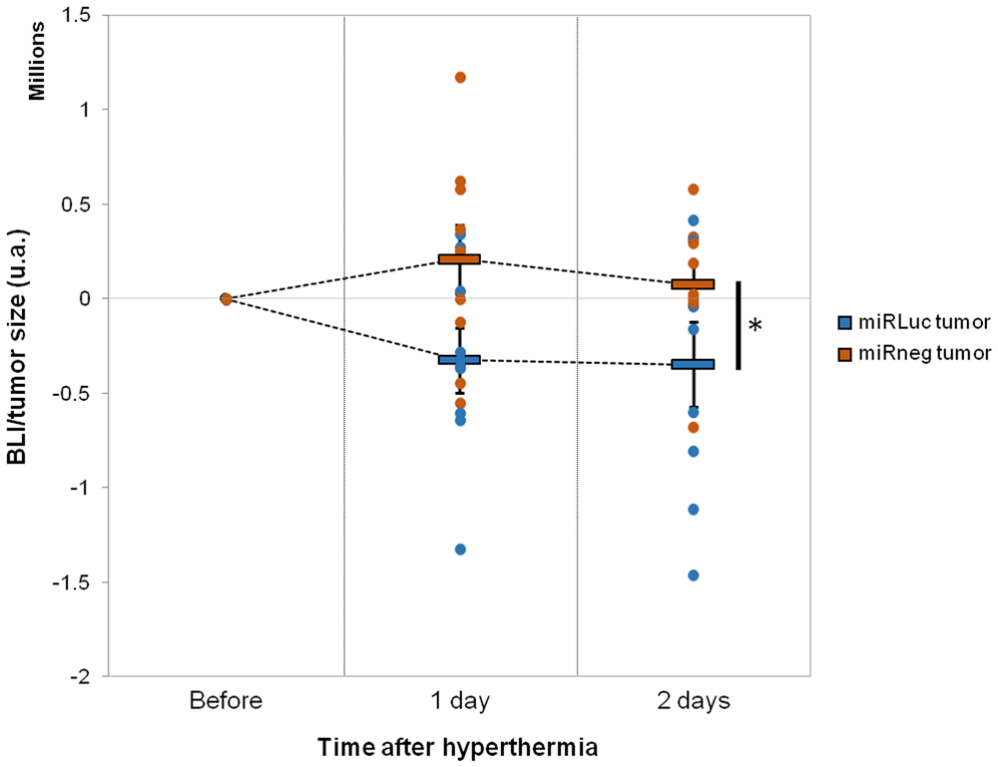
*In vivo* monitoring of thermo-induced silencing. Subcutaneous xenografts were generated by the injection of miRLuc or miRneg cell lines in mice hind legs. The BLI of LucF activity was measured in heat-induced miRLuc and miRneg tumors. The graph represents the BLI/tumor size ratio after hyperthermia corrected to before heat induction (n = 9 per group) (Student’s t-test between the two groups on the area under the curve, *p < 0.05).

To generate xenograft tumors, miRLuc and miRneg cell lines were injected subcutaneously in immunodeficient mice legs. The constitutive expression of LucF enabled tumor growth monitoring by BLI. *In vivo* hyperthermia was performed by bathing mice legs in a water-bath (45 °C; 8 min). LucR expression was then monitored to control hyperthermia efficiency (Fig. 3A). In accordance with our *in vitro* observation, the LucR bioluminescent signal was detected 6 hours after hyperthermia in both tumors. Synthetic miRNA contents and Hsp mRNA were quantified by qRT-PCR in tumor samples at different time points after hyperthermia (Fig. 3B and Supplementary Figure S6 respectively). The profile obtained for Hsp mRNA expression after hyperthermia *in vivo* was transient with a maximum at 6 hours as observed *in vitro* (Supplementary Figure S5) for the two cell lines. Furthermore, our data showed the induction of the synthetic miRNAs expression *in vivo*. These results confirmed the suitability of our heating method to induce Hsp70B promoters *in vivo*. Previous studies by others in mammalian cells showed a half-life of LucF of 3 hours^19,20^ and we previously measured a half-life of around 20 hours in an *in vivo* mouse model^21^. It is important to note that this half-life enables the measurement of any dynamic changes in LucF reporter transcription level *in vivo*. Subsequently, the effectiveness of miRLuc to reduce LucF expression was investigated by BLI monitoring. The time courses of LucF activity as well as the measurement of tumor growth were followed on both miRLuc and miRneg tumors from day one (beginning of the effect observed *in vitro*; Fig. 2B) to two days after hyperthermia. The BLI/tumor size ratio between the different time points after hyperthermia was calculated for miRneg and miRLuc tumors (Fig. 4). Our data shows a significantly higher reduction of the BLI/tumor ratio for the miRLuc tumor compared to miRneg after hyperthermia. This result corroborated our first *in vitro* observations and showed, *in vivo*, the efficiency of the heat-induced miRNA on its target gene.

## Conclusion

This study describes the utilization of a Hsp-controlled RNAi system in which a conditional synthetic miRNA was specifically designed to silence a reporter gene *in vivo*. We demonstrated the feasibility of on demand heat-triggered gene inhibition to ensure spatio-temporal control of gene inhibition *in vivo*. This method represents a new strategy that experts from different fields could use to address biological questions from their specific topics. For example, in the cancer field, it is well established that cell transformation is caused by multiple gene dysregulation (e.g. oncogenes). In such a pathological context, this new method could demonstrate its applicability to cancer treatment by targeting specific oncogenes in patient tumors. Indeed, we showed that several Hsp-driven transgenic expressions could be induced simultaneously including a mix of genes (two in the present work) and miRNAs (currently 4 copies) providing a way for multiple targeted regulation. But to achieve this goal, strategies will have to be developed to efficiently and specifically deliver to the tumor such synthetic miRNAs. The transient nature of our new method associated with engineered viruses or naked acid nucleic approaches may be promising to achieve gene inhibition while minimizing systemic toxicity. Finally, for this method, the activation of the Hsp70B promoter *in vivo* was accomplished using a water-bath which conferred the advantage of being a simple and low-cost system to induce hyperthermia on the entire tumor. More clinically relevant heating sources such as focal magnetic resonance-guided high-intensity focused ultrasound^10,16^ could be adapted to the Hsp-controlled RNAi system to reach deep tissues and to have a more precise spatial control.

The method demonstrated in this study offers multiple opportunities in terms of application, while also proposing a useful tool to measure efficiency of the inhibitory action of specific miRNA(s) on tumor cell proliferation monitored by BLI.

## Electronic supplementary material

Supplementary Information
