# Supplementary material for: Imaging of conditional gene silencing in vivo using a bioluminescence-based method with thermo-inducible microRNAs
Source: Sci Rep. 2018 Mar 16;8:4694. doi: 10.1038/s41598-018-22932-3 (PMC5856835; doi:10.1038/s41598-018-22932-3)
Supplement: Supplementary file 1 — Supplementary Information [file 41598_2018_22932_MOESM1_ESM.pdf]

Imaging of conditional gene silencing in vivo using a bioluminescence-based method with thermo-inducible microRNAs

Karine Pinel[1,#,*], Coralie Genevois[1,#], Christelle Debeissat[1] and Franck Couillaud[1,*].

SUPPLEMENTARY TABLES AND FIGURES

mRNA	Forwards primers	Reverses primers
LucF	5’- TCCATTCCATCACGGTTTTGG -3’	5’- GCTATGTCTCCAGAATGTAGC -3’
Hsp70	5’- CGCCTACTTCAACGACTCTCAG -3’	5’- GCTTGTTCTGGCTGATGTC -3’
36B4	5’- GCTTCATTGTGGGAGCAGAC -3’	5’- CATGGTGTTCTTGCCCATCAG -3’

Supplementary Table S1. Primer sequences used for reporter genes qRT-PCR.

miRNA	Stem-loop RT primers
miRLuc	5’- GTCGTATCCAGTGCAGGGTCCGAGGTATTCGCACTGGATACGTGAAAC -3’
miRneg	5’- GTCGTATCCAGTGCAGGGTCCGAGGTATTCGCACTGGATACGGTCTCC -3’
RNU44	5’- GTCGTATCCAGTGCAGGGTCCGAGGTATTCGCACTGGATACGAGTCAG -3’

Supplementary Table S2. Stem–loop RT primer sequences used for miRNAs reverse transcription.

miRNA	Forwards primers	Reverse primer
miRLuc	5’- CTCGGGTAATTCAGCCCATAT -3’	5’- GTGCAGGGTCCGAGGT -3’
miRneg	5’- TCGGGAAATGTACTGCGCG -3’	
RNU44	5’- CCTGGATGATGATAGCAAATGC -3’	

Supplementary Table S3. Specific primers sequences used for miRNAs quantitative PCR.

```
5′    1  ATGGAAGACG CCAAAAACAT AAAGAAAGGC CCGGCGCCAT TCTATCCGCT GGAAGATGGA

     61  ACCGCTGGAG AGCAACTGCA TAAGGCTATG AAGAGATACG CCCTGGTTCC TGGAACAATT

    121  GCTTTTACAG ATGCACATAT CGAGGTGGAC ATCACTTACG CTGAGTACTT CGAAATGTCC

    181  GTTCGGTTGG CAGAAGCTAT GAAACGATAT GGGCTGAATA CAAATCACAG AATCGTCGTA
                               ¦¦¦¦¦¦¦¦¦ ¦¦¦¦¦¦¦¦¦¦ ¦¦
                          3′-A CTTTGCTATA CCCGACTTAT GT-5′

    241  TGCAGTGAAA ACTCTCTTCA ATTCTTTATG CCGGTGTTGG GCGCGTTATT TATCGGAGTT

    301  GCAGTTGCGC CCGCGAACGA CATTTATAAT GAACGTGAAT TGCTCAACAG TATGGGCATT

    361  TCGCAGCCTA CCGTGGTGTT CGTTTCCAAA AAGGGGTTGC ... 3′
```

Supplementary Figure S4. Partial LucF coding sequence showing hybridization site of mature miRLuc.

The 21 nucleotides of the mature miRLuc (in bold) perfectly complementary hybridizes to the target sequence from nucleotides 200 to 220 of the LucF coding region.

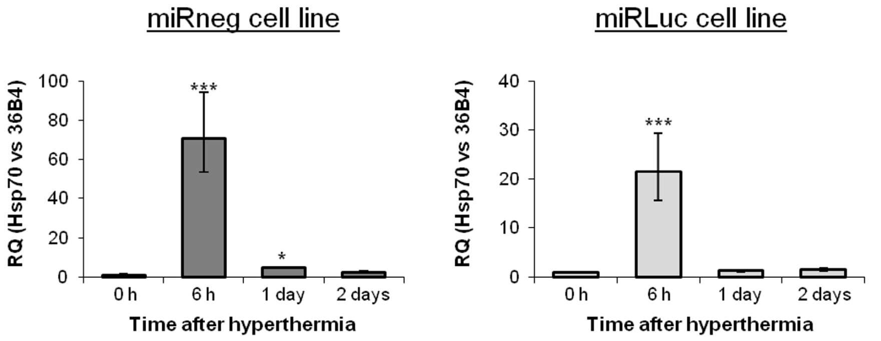

Supplementary Figure S5. *In vitro* heat-shock efficiency.

Relative quantification (RQ) of mRNA hsp70 determined by qRT-PCR with a maximum at 6 h post-hyperthermia in both miRLuc and miRneg cell lines (n=3) (Student’s *t*-test, *p<0.05 and ***p<0.001: *vs.* 0 h).

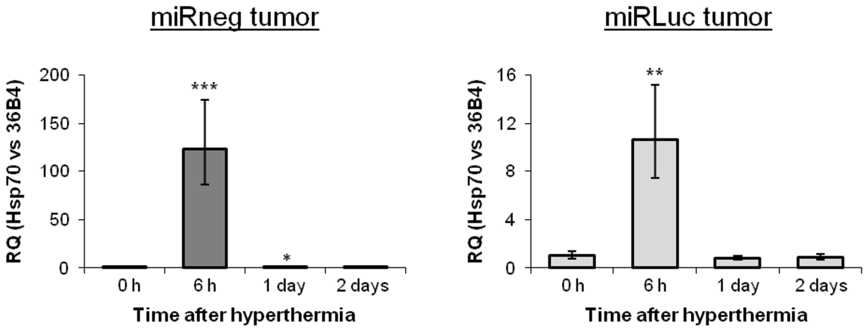

Supplementary Figure S6. *In vivo* heat-shock efficiency.
Relative quantification (RQ) of mRNA hsp70 determined by qRT-PCR in tumor samples which showed a maximum of expression at 6 h post-hyperthermia in both miRLuc and miRneg cell lines (n=3) (Student's *t*-test, *p<0.05, **p<0.01 and ***p<0.001: *vs.* 0 h).
